# Oak stands along an elevation gradient have different molecular strategies for regulating bud phenology

**DOI:** 10.1186/s12870-023-04069-2

**Published:** 2023-02-23

**Authors:** Gregoire Le Provost, Céline Lalanne, Isabelle Lesur, Jean-Marc Louvet, Sylvain Delzon, Antoine Kremer, Karine Labadie, Jean-Marc Aury, Corinne Da Silva, Thomas Moritz, Christophe Plomion

**Affiliations:** 1 INRAE, Univ. Bordeaux, BIOGECO, F-33610 Cestas, France; 2 Helix Venture, F-33700 Mérignac, France; 3 Genoscope, Institut François Jacob, CEA, Université Paris-Saclay, Evry, France; 4 Génomique Métabolique, Genoscope, Institut François Jacob, CEA, CNRS, Univ Evry, Université Paris-Saclay, 91057 Evry, France; 5 Department of Forest Genetics and Plant Physiology, Umeå Plant Science Centre, Swedish University of Agricultural Sciences, 901 87 Umeå, Sweden

**Keywords:** Response to temperature, Bud phenology, Elevation cline, Gene expression, Hormone quantification, Sessile oak

## Abstract

**Background:**

Global warming raises serious concerns about the persistence of species and populations locally adapted to their environment, simply because of the shift it produces in their adaptive landscape. For instance, the phenological cycle of tree species may be strongly affected by higher winter temperatures and late frost in spring. Given the variety of ecosystem services they provide, the question of forest tree adaptation has received increasing attention in the scientific community and catalyzed research efforts in ecology, evolutionary biology and functional genomics to study their adaptive capacity to respond to such perturbations.

**Results:**

In the present study, we used an elevation gradient in the Pyrenees Mountains to explore the gene expression network underlying dormancy regulation in natural populations of sessile oak stands sampled along an elevation cline and potentially adapted to different climatic conditions mainly driven by temperature. By performing analyses of gene expression in terminal buds we identified genes displaying significant dormancy, elevation or dormancy-by-elevation interaction effects. Our Results highlighted that low- and high-altitude populations have evolved different molecular strategies for minimizing late frost damage and maximizing the growth period, thereby increasing potentially their respective fitness in these contrasting environmental conditions. More particularly, population from high elevation overexpressed genes involved in the inhibition of cell elongation and delaying flowering time while genes involved in cell division and flowering, enabling buds to flush earlier were identified in population from low elevation.

**Conclusion:**

Our study made it possible to identify key dormancy-by-elevation responsive genes revealing that the stands analyzed in this study have evolved distinct molecular strategies to adapt their bud phenology in response to temperature.

**Supplementary Information:**

The online version contains supplementary material available at 10.1186/s12870-023-04069-2.

## Background

Despite extensive gene flow between populations [1], tree species display clear adaptation to local environmental conditions [2–4]. Ample evidence of the efficiency of natural selection can be found in the large body of literature on forest tree provenance tests (reviewed by [5, 6]. In almost all tree species for which provenance tests have been performed, significant genetic variation between populations (i.e. differentiation) has been observed for fitness-related traits, including bud phenology [7]. These common garden experiments have also underpinned clinal patterns of phenotypic variation along temperature and/or photoperiod gradients. European white oaks illustrate how diversifying selection generated clinal variation of bud phenology variation and the rapidity with which forest tree populations became locally adapted over a short time span following the end of the last ice age [8, 9]. Indeed, extant sessile (*Quercus petraea* (Matt.) Liebl.) and pedunculate (*Quercus robur* L.) oak populations originating from the same or different (refugial) sources of glacial origin, but currently growing at different latitudes display strong phenotypic differentiation for the timing of leaf unfolding [10], but no differentiation for neutral genetic markers [11, 12]. The same holds true for populations distributed along elevation clines [10, 13]. Divergent selection along temperature gradients drives adaptation for early-late flushing dates along low–high elevation (or latitude) to avoid late frost damages [13, 14].

Environmental gradients (with continuous variations of environmental conditions) impose spatially variable selective pressures on populations within the range of the species, inducing genetic differentiation. The distribution of genetic diversity across environmental gradients provides information about the effects of past selection on the genetic composition of populations and facilitates the identification of parts of the genome that have undergone selection [15, 16]. Although elevation gradients can present significant climatic variations (i.e. precipitation, cloud cover, irradiance, light quality), such gradients offer ideal opportunities for the discovery of genes of importance for adaptation to temperature: (i) phenotypic divergence between populations is generated by a predominant environmental driver — temperature — avoiding confounding effects with other potential drivers of adaptation, (ii) extensive gene flow between populations leaves the genome with narrow genomic windows in which spatially varying selection is maintained and detectable, and (iii) the populations studied share the same evolutionary history, preventing confounding contributions between demographic dynamics and natural selection. Elevation gradients have, therefore, been widely used to identify polymorphisms subject to natural selection [17], in diverse species, including forest trees [10, 18].

In the wild, Gene expression data have also proved powerful for the characterization of functional genetic variation and phenotypic plasticity between populations [19]. Comparisons of differences in expression profiles between locally adapted populations have been performed in many biological systems and have led to the discovery of genes of adaptive significance (reviewed by [20]). Moreover, the combination of allelic variation and gene expression data has proved very powerful for identifying signatures of adaptation in natural populations [21, 22]. However, it should be kept in mind that gene expression analysis in locally adapted populations to identify genes that matter for adaptation may have some limitations. Indeed, there is a shortcut between their differential expression and a clear sign of local adaption due to the possibility of epigenetic effects or possible population acclimation on gene expression. In Norway Spruce, [23] reported strong epigenetic effects on the expression of genes involved in bud phenology, cold acclimation and embryogenesis. Thus, genes identified by RNaseq must be validated by other approaches such as those described by [16].

As indicated above, bud phenology is a key trait contributing to local adaptation of forest trees. In temperate regions, the phenological cycle of the primary meristem can be split into two principal phases: a growing period (i.e. from bud burst to bud set), during which environmental conditions are favorable, and a resting period, also known as dormancy, from bud set to bud burst, when environmental conditions are unfavorable for stem elongation [24]. Dormancy begins when growth stops in late summer, in response to decreases in photoperiod and temperature [25], a phase known as paradormancy or summer dormancy [26]. This initial phase is followed by endodormancy, which is initiated by further decreases in temperature and photoperiod in the fall [27]. Endodormancy is generally associated with a cold acclimation process [28] and its induction is progressive, culminating in a complete lack of bud growth response under favorable conditions [29]. Endodormancy is the deepest stage of dormancy and is not released until chilling requirements have been fulfilled [30], generally around late January for oak populations in the Pyrenees [31]. In temperate regions, endodormancy generally starts in the early fall and peaks in early winter, with a timing dependent on the species considered and climatic conditions [32]. Once chilling requirements have been fulfilled, there is a transition from endodormancy to ecodormancy in the buds [24]. The development of ecodormant buds can be triggered by forced increases in air temperature in late winter and early spring. The timing of the phenological events observed in trees is correlated principally with photoperiod (the main factor triggering entry into endodormancy) and temperature (with chilling required to break endodormancy and increased temperature triggering ecodormancy release) [33]. These two environmental cues proved to be the most accurate in predictive models of leaf unfolding [34].

In the ongoing context of global warming (which is particularly pronounced in mountainous areas [35, 36]), serious concerns have been raised about the long term persistence of locally adapted populations at their current locations. Indeed, with increasing temperatures, the phenological cycle of tree species may be strongly affected, because higher winter temperatures may prevent endodormancy release if chilling requirements are not met during winter [31], and late frosts in spring may result in damage to trees if their buds flush too early [37]. Many molecular studies have been performed in trees, to decipher the molecular mechanisms involved in dormancy induction and release. These studies have revealed that dormancy is regulated principally via molecular pathways relating to phytohormones [38, 39], carbohydrates [40], temperature [41], photoperiod [42], oxygen species [43], water [44] and cold acclimation. Most of the studies aiming at deciphering the molecular players underlying the dormancy phases have focused on poplar [29], oak [45], spruce [46], chestnut [47] and beech [42]. However, these studies involved the use of a small number of genotypes harvested only during endodormancy or ecodormancy, and therefore provide a limited view of the internal and external drivers of dormancy induction and release. The most detailed study performed to date was published in poplar [48]. These authors identified key molecular pathways involved in bud formation and endodormancy induction and release. They also identified a large set of genes commonly expressed during growth-to-dormancy transitions, in poplar apical buds, cambium, or *Arabidopsis thaliana* seeds, suggesting that similar molecular mechanisms may underlie these processes in different plant organs. This study was however conducted under controlled temperature and photoperiod conditions. Moreover, none of the studies cited above used population genetics approaches to account for natural variation in the ability of the trees to adapt their phenological cycles to fluctuating external cues.

Here, we used sessile oak populations sampled along an elevation gradient in the Pyrenees to explore the gene expression network involved in dormancy regulation in this species. We addressed three main questions: Does gene expression in vegetative buds reflect divergent selection forces shaping population adaptation to elevation? Have stands sampled along the elevation gradient evolved different molecular mechanisms for regulating the endodormancy and ecodormancy stages? What gene functions (i.e. molecular mechanism) have served as targets for adaptation at the level of gene expression? We harvested terminal buds from trees growing at different elevations, during endo- and ecodormancy. The availability of long term records of flushing dates along the gradient allowed to account for differences in bud burst date between populations. We quantified gene expression by RNAseq and used a likelihood ratio test to identify genes displaying significant dormancy, elevation or dormancy-by-elevation interaction effects. The analysis of dormancy-by-elevation interaction effect is of main interest because this effect highlight different molecular strategies in the oak stands sampled along the gradient for adapting their bud phenology in response to environmental variation (i.e. temperature, in this study).

## Results

### Differences in phytohormone concentrations between elevations and dormancy stages

The endogenous concentrations of three main phytohormones (indole-3-acetic acid (IAA), abscisic acid (ABA) and cytokinin (CTK)) were determined on three biological replicates for each dormancy stage, population and valley. We first performed an ANOVA on the whole dataset, to determine whether the two valleys could be considered as biological replicates. We used the following fixed-effects model: Y_ijk_ = µ + D_i_ + E_j_ + V_k_ + εi_ik_, where D_i_ is dormancy stage (i = ”endodormancy” or “ecodormancy”), E_j_ is elevation (j = “low”, “medium” and “high” elevation) and V is the valley factor (k = “Ossau” or “Luz”). The results are shown in Additional File 1 Fig. S1 panel A. No significant valley effect was detected, as for the date of leaf unfolding (data not shown). In the second analysis, we therefore used the whole dataset, with the following fixed-effects model Y_ijk_ = µ + D_i_ + E_j_ + D_i_*E_j_ + ε_ij_, where D_i_ is the dormancy stage, and E_j_ the elevation effect, to identify significant dormancy, elevation and dormancy-by-elevation interaction effects. The results are summarized in Additional File 1 Fig. S1, Panel B for the three phytohormones analyzed.

IAA were higher in EcoD samples (i.e. ecodormant buds) at all elevations. CTK content were higher in EcoD samples only at low and high elevations, respectively. Finally, ABA concentrations were lower in EcoD samples at all elevations (Additional File 1 Fig. S1, Panel B). These results are consistent with those obtained by [49] and [50] for ecodormant poplar and endodormant pear buds, respectively, where similar hormone dynamics results were obtained over the winter period.

A dormancy-by-elevation interaction effect was identified for both IAA and ABA (Additional File 1 Fig. S1, Panel B). IAA concentration increased with elevation in EndoD samples (i.e. endodormant buds), but the opposite pattern was observed in EcoD samples. ABA concentration increased with elevation in EndoD samples, but remained stable across the cline in EcoD samples.

### RNA-seq analysis and identification of DEGs

The main goal of our study was to identify genes involved in dormancy regulation from the analysis of oak stands sampled at different elevations and potentially locally adapted to temperature (see methods section). We generated 24 cDNA libraries for this purpose. A general overview of the libraries generated is shown in Additonal File 2 Table S1. More than 597 million reads in total were mapped onto the *Q. robur* reference genome. We made the choice to map our RNAseq reads on a closely related species (pedunculate oak) because currently, no reference genome is available for sessile oak. Mapping rates ranged from 66 to 82%. In total, 17,676 genes out off the 25,808 gene models available in the *Q. robur* reference genome were retained after quality-based filtering.

**Fig. 1:**
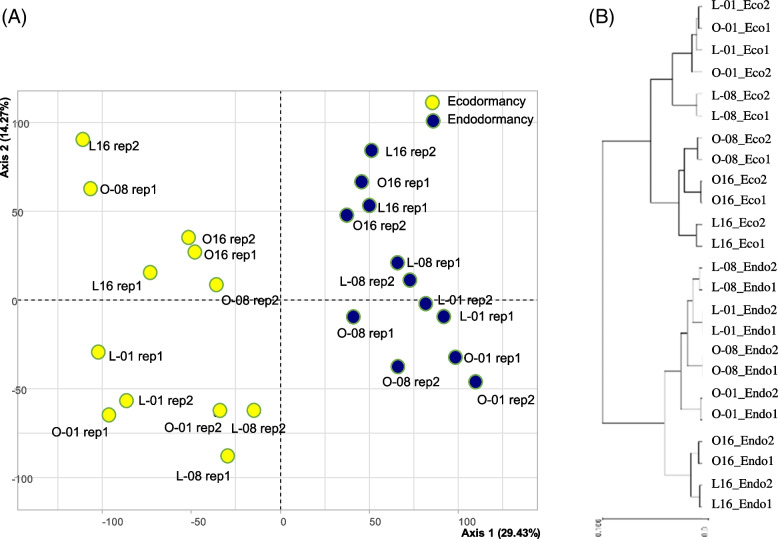
Evaluation of RNA-seq data quality using PCA (**A**) and clustering analysis (**B**) (using the “complete” method of the expender software) among the replicates of the different dormancy stages and elevations. For PCA blue and yellow dots represent EndoD and EcoD samples, respectively

We used PCA (Principal Component Analysis) to explore transcriptome-wide changes in our dataset. This exploratory analysis showed close clustering within biological replicates, supporting the quality of the RNAseq data (Fig. 1A). It also showed clustering of the samples into two distinct groups corresponding to each dormancy stage (Blue and Yellow dots spread along the first PCA axis in Fig. 1A). Finally, it should be noticed that elevations separated also along the PCA 2 axis within a dormancy stage (except for two replicates sampled at 800 m of elevation) suggesting a stronger influence of temperature rather than photoperiod on dormancy regulation in our experimental design.

The adjusted data were also evaluated by a clustering approach, with the complete method available in Expender software (Fig. 1B). This analysis confirmed the high reproducibility of the RNAseq data and revealed that, for each treatment analyzed in this study, the biological replicates and dormancy stages were grouped into the same cluster (Fig. 1B). Both the PCA (second PCA axis) and clustering analyses exhibited interactions between dormancy and elevation.

A preliminary analysis was performed to determine whether the two valleys could be considered as biological replicates for RNAseq. For each dormancy stage, we compared gene expression levels between pairs of population samples from the same elevation (i.e. O-01 vs. L-01, O-08 vs. L-08, O-16 vs. L-16), resulting in six different gene sets (2 dormancy stages * 3 elevations). The results are summarized in Additional File 2 Table S2. Regardless of the dormancy stage considered, we identified very few genes differentially regulated between the two valleys, the maximum proportion of DEGs reaching 1.2% of the 17,676 genes between O-08 and L-08 in EndoD samples. These results confirmed that the two valleys could be considered as biological replicates. Increasing the number of biological replicates (from 2 to 4) decreased the false-positive rate, facilitating the distinction between noise and true biological signals in our dataset.

**Fig. 2:**
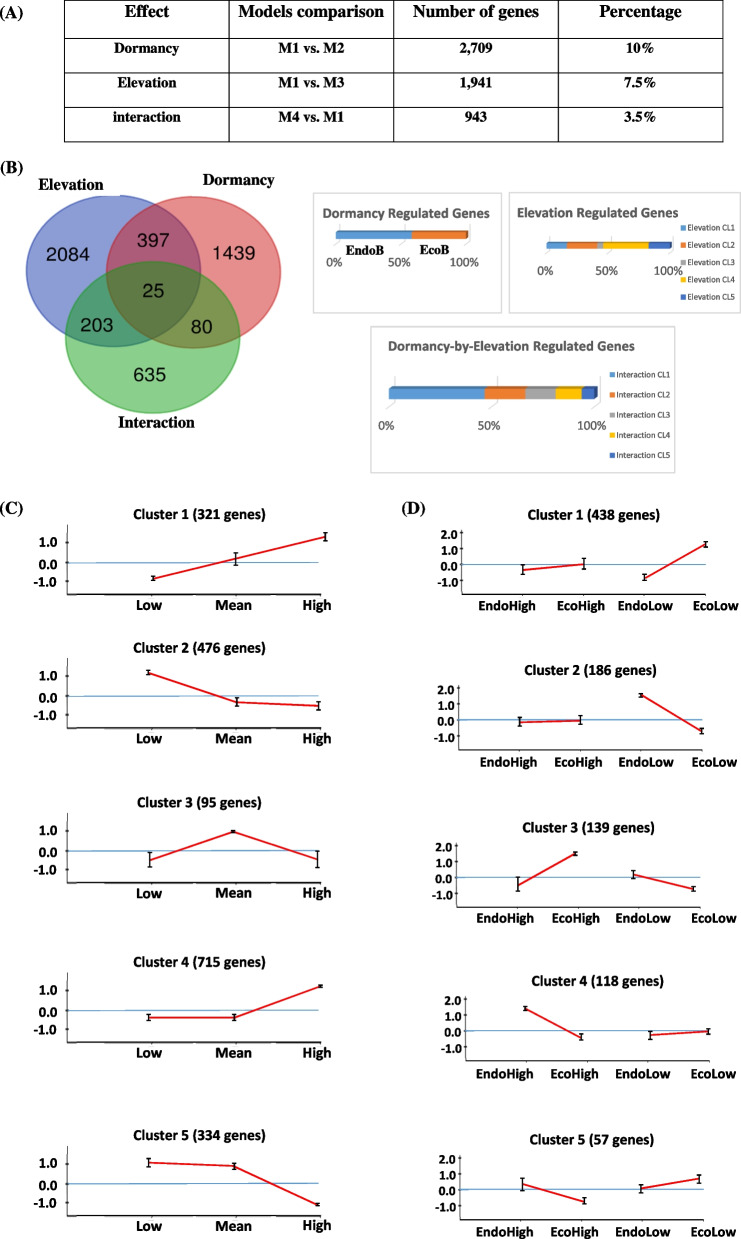
Illustration of the main results obtained from the DEGs analysis. Panel (**A**): Summary of the differential expression analysis. Results are shown for an adjusted *P*-value < 0.01 and a fold-change ratio > 2. The percentage indicated in the last column is given considering the 25,808 gene models found in the pedunculate oak genome. Panel (**B**): Venn-diagram showing the number of genes displaying main (Dormancy, Elevation) and dormancy-by-elevation-interaction effects or a combination of them. The Bar Chart illustrate the number of genes identified per effect and their associated clusters. Panel (**C**) Cluster identified for the elevation responsive genes and Panel (**D**) clusters identified for the dormancy-by-elevation responsive genes. For Panel (**C**) and (**D**) the X-axis represents the elevation (low: 100 m of elevation, mean: 800 m of elevation and high: 1,600 m of elevation. The Y-axis represents the normalized expression value for the genes belonging to the cluster (Mean and variance fixed to 0 and 1, respectively)

We then used likelihood ratio tests to identify genes displaying significant dormancy, elevation and interaction effects. Genes displaying differential expression (at least a two-fold difference in expression) with a corrected *P*-value < 0.01, and with adjustment for a false discovery rate (q-value) < 0.05 were considered to display significant differential expression. The results of the differential expression analysis are shown in Fig. 2, panel A. The overlap between the three effects is shown in Fig. 2, panel B. It should be noted that a very small number of genes (25) displayed all three effects simultaneously. Overall 2,084, 1,089 and 635 genes displayed significant dormancy, elevation and dormancy-by-elevation effects, respectively. We found that 397 genes were regulated by both dormancy and elevation, and that 203 genes displayed significant dormancy and dormancy-by elevation interaction effects (Fig. 2, panel B). Finally, 80 genes presented both significant elevation and dormancy-by-elevation interaction effects.

### Genes regulated during dormancy induction and release (Geneset#1)

By comparing models M1 and M2, using the threshold presented above, we identified 2,709 genes differentially regulated between EndoD and EcoD samples (Fig. 2, panel A, B and C). For this first gene set, most of the variation identified was quantitative (i.e. all the genes displaying differential expression were covered by reads from both the EndoD and EcoD libraries). We identified a single gene (Qrob_P0090160.2, encoding a riboflavin synthase) covered only by reads from EcoD samples. The results are presented in detail in Supplementary File 1. Gene set enrichment analysis was performed with the TopGo Package for this first gene set. Our analysis revealed different GO terms between EndoD and EcoD regulated genes. The results are shown only for the first 100 GO (ranked according to their *p*-value) in Supplementary File 1. A graphical representation (using bubble plots) of the first 20 ontologies is also available for a clearer view of the ontologies regulated.

We also performed subnetwork enrichment analysis independently for the genes regulated during EndoD and Ecod. We identified biological processes relating to cold tolerance, response to dehydration, freezing tolerance and drought tolerance in EndoD samples, whereas very different biological processes relating to meristem identity, flower development and floral organ identity were identified in EcoD samples. These results are consistent with those for dormancy induction and release reported in a previous study in oaks [45], validating the use of our experimental design and statistical model for finding dormancy-regulating genes and opening up promising new avenues for the identification of genes with expression profiles affected by elevation and interactions (see below).

### Elevation-regulated genes (Geneset#2)

By comparing models M1 and M3, we identified 1,941 genes regulated by elevation (Fig. 2, panel A, B and C and Supplementary File 2). We hypothesized that most of these genes may be involved in response to temperature. We clustered these genes according to their expression profiles. With the number of clusters set to 5, we obtained an overall homogeneity close to 0.98. Cluster#1 included 321 genes progressively upregulated by elevation; conversely cluster#2 contained 476 genes following the opposite pattern, whereas cluster#3 comprised 95 genes upregulated at intermediate elevations. Clusters #4 and #5 were strongly differentiated, with contrasting patterns of gene expression. Cluster#4 contained 715 genes that were overexpressed at the highest elevation, whereas cluster#5 contained 334 genes downregulated at highest elevation (Fig. 2, panel D and supplementary File 2).

We then performed gene set enrichment analysis on each cluster. GO terms enriched for each cluster in the three main ontologies are available in Supplementary File 2 both in tabular and graphical (bubble plot) representations. Subnetwork enrichment analysis was performed for each of the five clusters (Supplementary File 2). We identified hubs relating to leaf size, root growth and pollen development for the genes of cluster #1, senescence, cell death and cell division for cluster #2, root development, root growth and flower development for cluster#3, internode patterning, fatty acid omega oxidation and pedicel development for cluster #4, and heat tolerance for cluster #5.

### Genes displaying a significant dormancy-by-elevation interaction effect (Genest#3)

Using the likelihood ratio test to compare models M4 and M1, we identified 943 genes displaying a significant dormancy-by-elevation interaction effect (Fig. 2 panel A, B and D and Supplementary File 3). The majority of these genes displayed this effect only (635 of 943 genes, Fig. 2 panel B). Only 25 genes displayed all three main effects simultaneously. The remaining genes (*i.e.* 283 genes) displayed one of two significant effects: significant effects of dormancy for 203 genes and of elevation for 80 genes. We also applied the Kmeans method to this gene set to cluster genes according to their expression profiles. A stable homogeneity value of approximately 0.91 was obtained for a *k* value of five, giving five different clusters (Fig. 2 panel D and Supplementary File 3). Cluster #3 (139 genes) and cluster #2 (186 genes) had similar expression profiles characterized by a singular pattern in which certain genes were upregulated in Ecod samples at high elevation and in EndoD samples at low elevation. Cluster #1 (438 genes), cluster #5 (57 genes) and cluster #4 (118 genes) encompassed genes upregulated in EcoD samples at low elevation, but with stable or downregulated expression at the same developmental stage at high elevation.

GO terms enrichment analysis for each cluster is available in supplementary File 3 (both in tabular and graphical representations).

The subnetwork enrichment analysis is also shown for each cluster in Supplementary File 3. For cluster #2 and cluster #3, which were characterized by genes upregulated in EcoD samples in populations growing at high elevation, we identified significant enrichment for biological processes relating to the stress response: genes relating to “salinity”, “plant defense” and “plant development” in cluster #2, and genes involved in “salinity response”, “detoxification” and “cell elongation” in cluster #3. For clusters #1, #4 and #5, characterized by genes upregulated in EcoD samples in populations growing at low elevation, most of the biological processes identified were related to meristem functioning. Important hubs relating to “plant growth”, “mRNA splicing” and “transcription activation” were identified in cluster #1. For the other two clusters (#4 and #5), we identified hubs relating to “plant growth”, “flower development” and “seed germination” for cluster #4, and “cell elongation”, “developmental process” and “flower development” for cluster #5.

### RT-qPCR validation

In total, 12 genes were selected for validation of their expression profiles by real time reverse transcription-quantitative PCR. Two of these genes displayed either a multi-banding pattern on agarose gel electrophoresis or unsuccessful PCR amplification and were removed from the analysis. For the other 10 genes, PCR efficiency ranged from 95 to 110%, in accordance with *Taq* polymerase activity (Additional File 2 Table S3). The patterns of expression of the genes tested by RT-qPCR were similar to those obtained by RNAseq (Additional File 1 Fig. S2), validating our RNA-seq data.

## Discussion

The main goal of this study was to provide new insights into the key molecular pathways involved in dormancy regulation. We used sessile oak populations sampled along an elevation gradient in the Pyrenees to explore the gene expression network involved in dormancy regulation in this species.. We considered the two main phases of dormancy — endodormancy and ecodormancy — and performed an analysis of differential gene expression on terminal vegetative buds by an RNAseq approach. We identified genes displaying significant changes in expression associated with dormancy, elevation and dormancy-by-elevation interaction. Our results highlighted a number of molecular processes at work in sessile oak populations along an elevation cline. Our findings provide also a set of candidate genes involved in the response of sessile oaks to temperature at different elevations. Investigating genomic signatures of local adaptation in and around these loci could help identify the genetic bases of adaptation along this elevation gradient. Further, inform predictive models aiming to infer the risk of maladaptation in assisted migration strategies in the framework of global warming [16].

The variation of dormancy-related traits has been well investigated along latitudinal clines, in which both photoperiod and temperature vary [51, 52], but elevation clines over a restricted area have major advantages for disentangling these two environmental factors and provide a unique opportunity to analyze the effect of temperature independently from that of photoperiod [9]. Moreover, to our best knowledge, elevation clines have never been used to analyze the molecular mechanisms involved in bud dormancy regulation in a forest tree species. The predicted temperature increase in the coming years can be expected to affect the phenological cycle of perennial species [28, 37]. We therefore mainly focused the discussion on genes regulated by elevation (i.e. temperature-responsive genes) and genes displaying a significant dormancy-by-elevation interaction effect, because these two sets of genes may include genes of importance for forest tree adaptation. However, we have to bear in mind that the molecular mechanisms identified in this study were obtained using a single sampling campaign. Dormancy induction and release is strongly influenced by temperature and photoperiod. Thus, interannual climate variability can influence gene expression, leading potentially to different gene networks identified if multi-year sampling campaigns had been used.

### Molecular plasticity during development underlying the shift between endo- and ecodormancy

We first quantified three phytohormones (IAA, ABA and cytokinines) to characterize the samples phenotypically. As reported in fruit trees [53], ABA content was higher in EndoD samples. Several authors have also reported a role for ABA in the photoperiodic control of growth and, potentially, in the cessation of growth induced by short days [29]. Conversely, IAA and cytokinin concentrations were higher in EcoD samples. IAA concentration is known to increase during the reactivation of cambium activity [50] and cytokinins are activated during budburst [54]. These findings suggest that the higher concentrations of these two phytohormones in the buds during ecodormancy are correlated with the reinitiation of mitotic activity in the buds of the sampled trees.

Transcriptomic studies identified two biological networks corresponding to gene set#1 related to either EndoD or EcoD samples (Supplementary File 1). As expected, important hubs related to cold tolerance, freezing tolerance, drought tolerance and defense response were identified in the EndoD network, whereas biological hubs related to meristem functioning (meristem identity, flower development, floral organ identity, etc.) were found in the EcoD network. Similar results have already been published for oak [45] and beech [42], but the dataset reported here is much broader than those previously published, extending to genes with lower levels of expression (e.g. genes encoding transcription factors). The upregulation of molecular mechanisms involved in dehydration and cold tolerance during endodormancy is consistent with the greater tolerance to ice formation of cold-tolerant dehydrated tissues, making it easier for the plant to cope with cold winter temperatures [55]. The central hubs (i.e. biological processes) relating to cell activity and meristem functioning identified in the ecodormancy network reflect the reinitiation of the cellular metabolism required for bud burst when environmental conditions become favorable [56, 57].

### Environmental molecular plasticity in response to temperature

In mainland France, regardless of the climatic scenario modeled, mean temperature is predicted to increase by 0.6 °C (in northern France) to 1.3 °C (in southern France) by 2050, with even greater temperature contrasts predicted for mountainous areas [58]. This increase in temperature may influence the distribution of forest ecosystems [59]. Thus, the elevation cline used here, with a temperature gradient extending over 6.9 °C, provided a opportunity to analyze the role of gene expression in responseto temperature.

#### (a) Populations from low and medium elevations

**Fig. 3:**
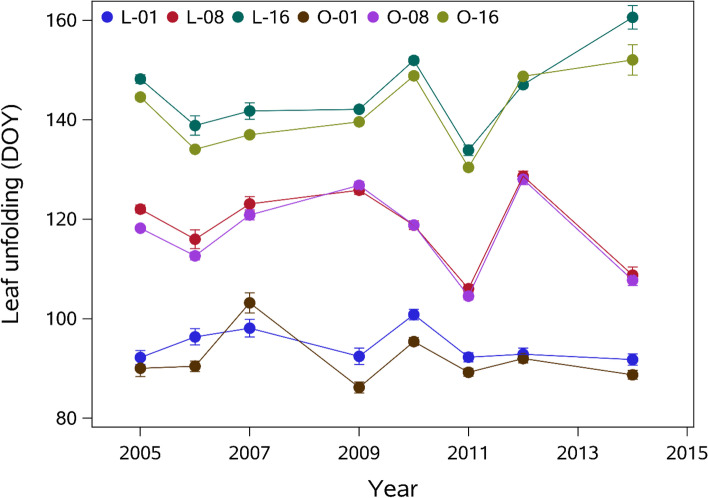
Mean date and standard error of leaf unfolding (y-axis, Date of the Year) for the three selected sessile oak populations in the Luz (L) and Ossau (O) valley at 100 (O-01, L-01), 800 (O-08, L-08) and 1,600 m (O-16, L-16) a.s.l

Two clusters encompassed genes upregulated at low and/or medium elevations (Cluster #2 and #5, respectively, Supplementary File 2, Fig. 2 panel C). Populations of these two clusters exhibit an extended growing season characterized by a very early budburst date (Fig. 3). This phenology may be explained by the main molecular mechanisms identified in our subnetwork enrichment analysis.

First, we identified a large number of genes in Cluster #2 involved in cell death inhibition, suggesting an extension of cell life expectancy, which would enable the tree to extend its growing period. Indeed, half the genes relating to the cell death biological process are involved in cell death inhibition (CAS1, BON3, GLIP1, TOPII, T3G21.18 and F5O11.34). For example, we identified a CAS1 gene encoding a cycloartenol synthase 1 (4.2 fold-change ratio -FC- between low and high elevation populations). Kim et al*.* (2010) [60] showed that *Arabidopsis* transgenic lines in which this gene was downregulated were characterized by a cell death phenotype, suggesting a key role of the CAS1 gene in cell death inhibition. Similarly, *BON3* (3.8 FC), a gene encoding a copine-like protein, has been reported to be involved in inhibiting cell death [61]. A TOPII gene (2.24 FC) encoding a topoisomerase-like protein, was found to be downregulated in rice, this downregulation being associated with delayed cell death [62]. We also identified 15 genes encoding proteins involved in the biological process “senescence”, including five genes (MYB62 (2.45 FC), AGL15 (4.35 FC), LOX2 (2.15 FC), ABI5 (2.81 FC) and SAG13 (2.23 FC)) involved in the inhibition of senescence, in agreement with our observations indicating that leaf fall occur later in populations growing at lower elevations. For example, the MYB62 gene, which encodes a R2R3 MYB transcription factor, is known to be involved in apical dominance, delayed flowering time and late senescence in sunflower [63]; and the AGL15 gene, encoding an agamous-like protein, has been clearly implicated in delayed senescence in *Arabidopsis thaliana* [64].

Finally, nine genes involved in cell division and 23 genes involved in root growth were identified, suggesting that populations growing at lower elevations may have higher levels of meristematic activity, enabling the trees to flush earlier, thereby extending their growing period. One of these genes, *ERF3,* encodes a protein similar to an ethylene-responsive factor that stimulates cell division in the root tip in rice [65]. Another, *MYB12,* encodes a protein known to promote cell division and elongation in *Arabidopsis* [66].

Cluster #5 was enriched in a single biological process related to “heat tolerance” (Supplementary File 2). All the genes in this hub are involved in the acquisition of heat tolerance. This mechanism enables higher plants to cope with heat stress and to maintain their productivity under unfavorable environmental conditions (reviewed by [67]). We hypothesized that higher heat tolerance acquisition in populations growing at lower elevations would be an effective strategy for coping with stressful conditions more frequently encountered in such populations, which have an extended growing period. Two of the genes identified were considered to be of particular importance: *AGO1* (FC = 2.26) and *TOR* (FC = 2.55). *AGO1* encodes an RNA slice protein known to be essential for the maintenance of acquired heat tolerance [68], and the downregulation of TOR (encoding a phosphatidylinositol 3-kinase family-like protein, TOR, AT1G50030) in the shoot apical meristem of *Arabidopsis thaliana* is known to weaken heat tolerance [69].

#### (b) Populations from high elevations

Two other clusters (#1 and #4) contained genes upregulated at higher elevations (Supplementary File 2, Fig. 2 panel C). Oak populations from high elevations flush later and their vegetative phase is much shorter, occurring when environmental conditions are favorable [9]. Moreover, leaf size is smaller in high-elevation than in low-elevation populations [70]. The molecular mechanisms we identified in the subnetwork enrichment analysis may explain these observations. Indeed, biological processes relating to “leaf size”, “pollen development”, “root growth”, “internode patterning” and “pedicel development” displayed enrichment in clusters #1 and #4.

Ten of the genes in cluster #1 are involved in the biological process “leaf size”. Three of these genes (POL, T1M15.220 and GAI) have been implicated in the reduction of leaf size, the others being essential for normal leaf development. POL (FC = 2.47 between high- and low-elevation populations) encodes a protein phosphatase 2C. It is known to be involved in reducing leaf size in *Arabidopsis* [71]. T1M15.220 (encoding a protein resembling SAUR, FC = 2.21) is also known to be associated with smaller leaf size in plants (reviewed by [72]). Finally, the overexpression *GAI* (encoding a GRAS family transcription factor, FC = 2.63) has been shown to lead to smaller leaf size in petunia [73].

Cluster #1 also included 16 genes relating to root growth and seven genes relating to meristem size. Most of the genes identified in this cluster are essential for meristem functioning, again illustrating the ability of high-elevation populations to produce leaves over a short growing period. For example, the BIG gene (encoding an auxin transporter protein, FC = 2.6) is known to be essential for cell proliferation in response to sucrose and glucose in *Arabidopsis* roots and shoot meristems [74]. RPK2 (encoding a protein resembling a receptor-like kinase RPK2, FC = 2.36) is known to be involved in regulating meristem size by controlling cell proliferation [75].

Eleven genes relating to the biological process “pollen development” were also identified, suggesting that the cellular machinery of high-elevation populations is activated to ensure rapid flushing and flowering when environmental conditions become favorable. These 11 genes included three GSL genes (GSL1 (FC = 2.32), GSL8 (FC = 2.49) and GSL10 (FC = 2.62)) encoding callose synthases. Callose synthase has been reported to play a key role in pollen development in *Arabidopsis thaliana* [76]. We also identified two RGL genes (RGL2 (FC = 2.77) and RGL1 (FC = 3.47)) encoding the DELLA protein. Fleet & Sun, (2005) [77] reported that the DELLA gene repressed petal and pollen development, together with anther elongation, in *Arabidopsis*, suggesting a possible role of these genes in the delay of flowering time observed in high elevation populations.

Finally, only one biological process related to “internode patterning” and corresponding to three genes (KNAT1 (FC = 2.63), STM (FC = 4) and BLH8 (FC = 2.43)) was identified in cluster#4 (Supplementary File 2). Two of these genes (STM and BLH8) control meristem formation and/or maintenance, organ morphogenesis, organ position, and several aspects of the reproductive phase in *Arabidopsis thaliana*, suggesting a possible key role in meristem function [78].

**Fig. 4:**
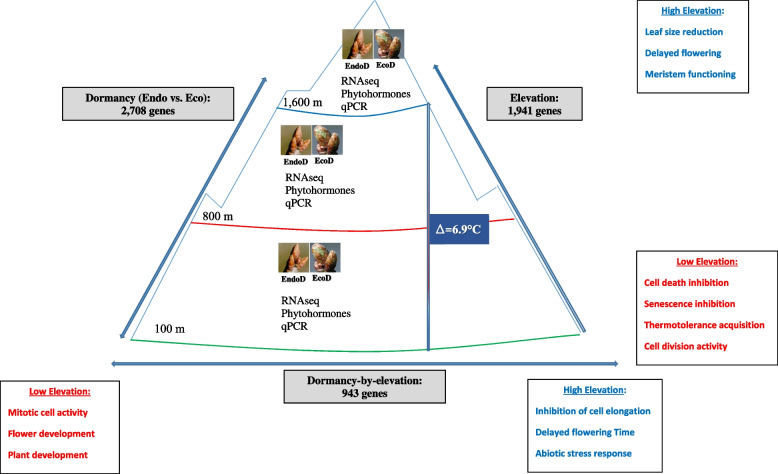
Overview of the results obtained in this study. Main molecular functions associated to elevation and dormancy-by-elevation interaction effect are indicated in color. Red color was used for population from low elevation, Blue color for population from high elevation. The number of genes displaying a significant (i) dormancy, (ii) elevation or (iii) dormancy-by-elevation interaction effect is indicated in grey

To summarize, our study indicates that the main molecular mechanisms associated with genes differentially expressed at low and high elevations may account for the typical phenology and features of adaptation to temperature observed in these oak stands. Indeed, at low elevation, populations display a higher basal level of expression for genes involved in the inhibition of cell death and senescence, the acquisition of heat tolerance and the maintenance of cell division activity, enabling the trees to flush earlier when environmental conditions are favorable (Fig. 4). Conversely, populations from high elevations are characterized by an overexpression of genes involved in leaf size reduction, meristem function and delayed flowering, enabling the trees to cope with the shorter growing season at high elevations (Fig. 4). It should be also noticed that our results are also in accordance with those of [79]. Indeed, these authors reported that at low elevation natural selection favors vegetative growth than reproduction thus explaining the early flushing date observed at low elevation.

### Dormancy-by-elevation interaction-responsive genes reveal different molecular strategies for coping with temperature variation in the oak stands

The five clusters identified with the Kmeans approach were merged into two main groups: a group of genes upregulated in ecodormant buds at high elevation (clusters #2 and #3, Fig. 2 panel D) and a group of genes upregulated in ecodormant buds at low elevation (clusters #1, #4 and #5, Fig. 2 panel D).

The high-elevation gene pool corresponds to late flushing populations [80]. These populations have to cope with severe environmental conditions. A 6.9 °C temperature gradient has been reported along the studied elevation cline. Once chilling recruitment has been fulfilled, the buds in the high-elevation populations enter the ecodormancy phase. However, they still have to cope with damage due to the late frosts known to occur during early spring at this elevation. The main molecular mechanisms identified in clusters #2 and #3 (*i.e.* flowering time, plant development, salinity response and detoxification (*i.e.* cold tolerance)) may explain the typical bud phenology observed in these high-elevation populations.

By contrast, populations at low elevations consist of early-flushing trees with a longer growing period [80]. We thus assume that the main biological processes found in clusters #1, #4 and #5 (cell activity, meristem functioning and flower development) prepare the buds to flush as soon as the heat requirement is fulfilled, accounting for the longer growing period observed in these populations.

An analysis of the genes displaying an elevation x dormancy phase interaction effect therefore provides elements of the answer to the following additional questions: how do populations at high elevation cope with late frost damage during ecodormancy? Which molecular mechanisms account for the early flushing date observed in populations at low elevation?

#### (a) How do populations from high elevations cope with adverse environmental conditions during ecodormancy to avoid late frost damage (cluster#2 and #3)

The network enrichment analysis performed for clusters #2 and #3 (Supplementary File 3) identified several genes involved in the inhibition of cell elongation and/or delayed flowering time, but also genes involved in the response to abiotic stresses. For the biological process “flowering time”, we identified ABF4 (a b-zip transcription factor) known to be involved in delaying flowering time in *Arabidopsis* [81]. The WRKY71 transcription factor, also found in cluster#2, directly affects the flowering time of plants by regulating the Constance and Flowering locus genes [82]. The CRY1 gene (cryptochrome 1 apoprotein) encodes a protein involved in the photoperiodic regulation of flowering and the inhibition of cell elongation [83]. MFB16.6 (encoding the SPL13 protein) was also upregulated during ecodormancy in high-elevation populations. Gao et al*.* (2018) [84] reported a potential role of this gene in delaying flowering time in *Medicago*. We also identified many genes involved in the regulation of cell elongation, generally through its inhibition, suggesting a potential role for these genes in slowing meristem development. Two of these genes, SWN and At3g51890, encode a swinger and the CLC3 protein, respectively. Footitt et al*.* (2015) [85] reported that peak SWN gene expression coincided with peak dormancy in *Arabidopsis*, suggesting a key role of this gene in dormancy release. Wang et al*.* (2013b) [86] identified the CLC3 gene as a key molecular player involved both in basipetal transport and the sensitivity and distribution of auxin. However, the principal role of CLC3 is in auxin transport, and it was difficult to relate its expression level to auxin content in our biological samples.

Moreover, cluster#3 contained genes potentially involved in the acceleration of cell elongation or in the activation of flowering, potentially accounting for the faster growth and more rapid reproduction of these populations. BZR1 (encoding a positive regulator of brassinosteroid signaling) is known to induce cell elongation at high temperatures, under the combined effect of auxin and brassinosteroids [87]. *ROT3*, encoding a cytochrome P-450, is known to be involved in determining the rate of leaf expansion in response to brassinosteroid [88]. We also identified a CIB1 gene encoding a protein similar to the cryptochrome-interacting-basic-helix-loop-helix protein. Liu et al*.* (2018) [89] showed that cryptochromes are blue light receptors that mediate light responses in plants and animals and activate flowering once environmental conditions are favorable (temperature and photoperiod).

Finally, we identified several genes relating to “salinity response”, “plant defense” and ‘detoxication”. This is not surprising, because these processes may enable the bud to remain dehydrated during ecodormancy, and, therefore, to cope more effectively with the low temperatures frequently observed during early spring at high elevations. Several studies have also reported that detoxication processes are essential for the acquisition of cold tolerance in plants [90, 91]. For example, we identified an APX3 gene encoding an ascorbate peroxidase involved in ROS detoxication. Wang & Li (2006) [92] reported that APX genes are overexpressed during cold acclimation. We also found an OSSA1 gene encoding a protein similar to a cytosolic O-acetylserine(thiol)lyase known to be involved in ROS detoxication in plants [93]. Two GSTU genes (GSTU17 and GSTU9) encoding proteins resembling glutathione transferase were also identified. Fujino & Matsuda, (2009) [94] showed that the GSUT gene contributed to cold tolerance in rice. The PR4 gene encodes a pathogenesis-related protein, and Cabello et al*.* (2012) [95] reported that the induction of this gene led to cold tolerance in *Arabidopsis*.

#### (b) Which molecular mechanisms underlie the early flushing observed in populations from low elevations?

Our subnetwork analysis on clusters #1, #4 and #5 (Supplementary File 3) highlighted biological processes relating to “mitotic cell activity”, “flower” and “plant development”. These molecular mechanisms are potentially involved in the early bud break observed in low-elevation populations and may account for the longer growing period of these populations. Genes relating to “plant growth” were mostly found in cluster#1 (32 genes). Seven genes with a potential role in the early initiation of meristem activity were identified: (i) a GR-RBP2 and two emb1138 genes encoding a glycine- and lysine-rich RNA-binding protein and DEAD box RNA helicases, respectively. Members of these two gene families are known to be involved both in cold stress tolerance and accelerating seed germination and seedling growth at low temperature [96, 97], (ii) an FBL17 gene encoding a protein similar to an ubiquitin protein ligase FBL17 was also detected. Noir et al*.* (2015) [98] showed that a loss of FBL17 function strongly decreased cell division rates in the apical meristem. Similarly, an SHR gene encoding a protein resembling GRAS family transcription factors, which known to be key regulators of cell division and meristem activity, was also identified [99], (iii) an SE gene encoding a C2H2 zinc-finger protein, Serrate (SE), which is known to accelerate leaf production and to decrease time-to-flowering, was also identified [100], and finally, (iv) we identified two genes (ARIA and F21P24.13) involved in ABA sensitivity, heat tolerance and seedling growth, suggesting a potential role of these genes in reinitiating meristem activity [101].

We also identified four and six genes relating to “flower development”. Notably, cluster#4 included a LOX3 gene and a GA20X2 gene involved in hormone signaling. The LOX3 gene encode a lipoxygenase implicated in the biosynthesis of jasmonic acid and flower development [102]. The GA2OX 2 protein resembles a gibberellin 2-beta-dioxygenase known to be involved in the gibberellin biosynthesis pathway. Yamauchi et al*.*, (2007) [103] reported a role for this gene in partly suppressing germination during exposure to darkness after phytochrome inactivation. An F15K9.22 gene encoding a protein similar to the Fantastic Four (FAF) protein was also found in this cluster. This gene influences the activity of the shoot apical meristem and is upregulated once flowering is initiated [104].

Cluster#5 included three genes (RAP2.7, GAI and LP1) with potential roles in both the early budburst and early flowering observed in populations from low elevations: (i) RAP2.7, encoding a protein resembling Apetala2 protein. Okamuro et al*.* (1997) [105] showed that this gene is active in the meristem during both reproductive and vegetative development, (ii) a GAI gene encoding a protein resembling a transcription factor involved in gibberellin signaling. In *Arabidopsis thaliana,* the upregulation of this gene accelerates flowering [106], and (iii) an LP1 gene encoding a non-specific lipid transfer protein. Nieuwland et al*.* (2005) [107] showed, by RNAi in *Arabidopsis*, that the LP1 gene played an important role in meristem function. Its downregulation resulted in dwarfing and a disruption of flower development.

## Conclusion

In summary, this study provides a comprehensive overview of the sessile oak bud transcriptome along an elevation gradient. Our monitoring of gene expression along altitude and during eco and endodormancy highlights + plastic responses to temperature, but also a potential genetic control of this plastic response. Indeed low- and high-altitude populations have evolved different molecular strategies for minimizing late frost damage and maximizing the growth period, thereby increasing their respective fitness in these contrasting environmental conditions. As summarized in Fig. 4, late budburst of populations at high elevation was found to be related to the overexpression of genes involved in the inhibition of cell elongation and delaying flowering time. Genes associated with cold tolerance were also found, enabling the buds to avoid late frost damage during early spring. Conversely, in populations at low elevation, we found a higher expression of genes involved in accelerating cell division and flowering, enabling buds to flush earlier once environmental conditions become favorable.

A molecular understanding of how oak stands have fine-tuned their leaf unfolding process to a temperature gradient throughout the dormancy period, over micro-evolutionary time scales, provides a valuable basis for further investigation in light of the increasing temperatures due to global warming. The candidate genes that we identified could be validated in the future by studying their gene expression level in reciprocal transplantations. Such experimental designs are available [70] for the analyzed stands and will be helpful in the coming years to quantify the respective contributions of phenotypic plasticity and genetic variation.

## Methods

### Study area and populations

**Fig. 5:**
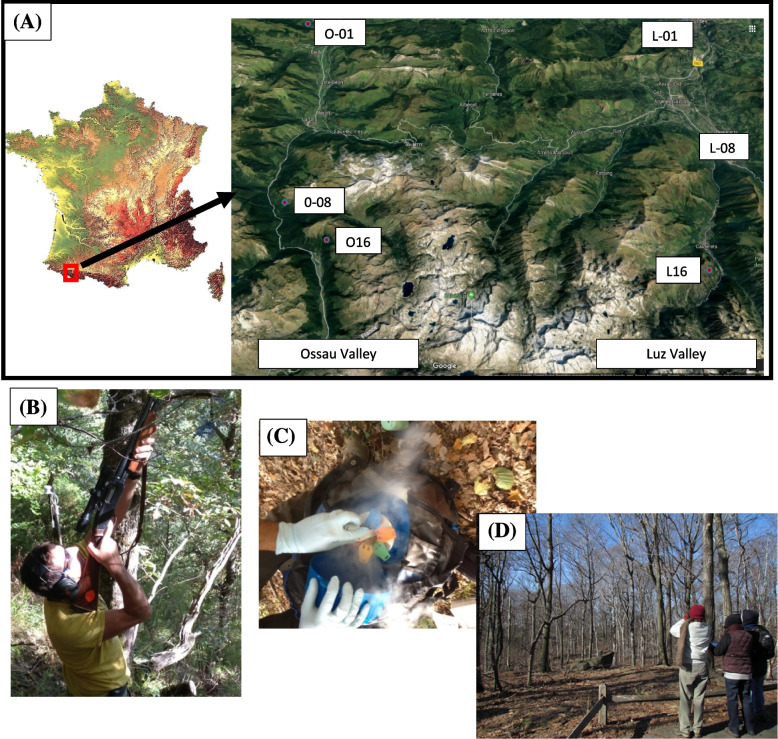
Sampling buds from sessile oak trees in the Pyrenees. (**A**): Location of the three selected populations along two elevation clines (Ossau and Luz valleys). Population ID follows that of Additional file 2 Table S4. (**B**): Shotgun to harvest terminal branches on three canopee, (**C**): Bud storage in liquid nitrogen, (**D**): Leaf unfolding observation with binoculars

We studied natural populations of sessile oaks (*Quercus petraea* (Matt.) Liebl.) growing in two neighboring valleys (Ossau and Luz, denoted O and L, respectively) on the northern side of the Pyrenees in France (from 43°15′N, 00°44′W to 42°53′N, 00°06′E). In each valley, populations grew at elevations varying between 100 and 1,600 m a.s.l., corresponding to a temperature gradient of 6.9 °C between the populations at the lowest and highest elevations [108]. Functional traits related to phenology, morphology and physiology of these populations have been extensively monitored in situ and ex situ (i.e. in common gardens) over the last 15 years, to quantify the respective contributions of phenotypic plasticity and genetic variation to their within and between population variation [13, 31, 70, 109]. In addition, reciprocal transplant experiments conducted at a smaller scale confirmed the co-gradient variation [6] of the timing of bud burst along elevation, thus enhancing local adaptation [80]. A general overview of the populations sampled in this study is provided in Additional File 2 Table S4 and Fig. 5A.

### Bud phenology monitoring and sampling

The timing of leaf unfolding was recorded over a period of eight years (between 2002 and 2014) in six populations, consistently taking measurements from the same 20–34 adult trees per population each year, over the two elevation gradients. Leaf unfolding was monitored at 10-day intervals in each population, from March to June, by the same two observers with binoculars (magnifying power × 10, Fig. 5D), standing about 15 m away from the tree. At each visit, the developmental state of each tree was assessed and the date of leaf unfolding relative to January 1^st^ (i.e. day of the year, DOY) was determined as the date at which the leaves of 50% of the buds were fully unfolded, as described in a previous study [9].

We selected three populations per valley, based on the timing of leaf unfolding, as representative of the intraspecific phenological variability along these two elevation clines (Fig. 3). These populations were growing 100, 800 and 1600 m a.s.l. and differed considerably in terms of the timing of leaf unfolding (about 50 days’ difference between the populations at the lowest and highest elevations).

Buds were sampled as follows: (i) a first sampling campaign was performed during the endodormancy period in the fall of 2013. Endodormancy is triggered by both light and temperature, but with photoperiod playing the dominant role [110], as demonstrated by short-day experiments [111]. We therefore harvested buds from all the trees during the same week. The samples collected were endodormant buds, referred to hereafter as EndoD buds, (ii) a second sampling campaign was performed in spring 2014, just before bud burst. As release from ecodormancy is driven primarily by increases in temperature (i.e. trees from populations growing at high elevations flush later than those from lower elevations), we used the leaf unfolding time series to determine the most relevant sampling dates at each elevation. Sampling thus spanned a period of four weeks, from early March to early April (Additional File 2 Table S4). The samples collected were ecodormant buds, referred to hereafter as EcoD buds.

Buds were sampled from the same 20 trees in each population for both developmental stages. A shotgun was used to detach the terminal branches for sampling. The terminal buds were then harvested and immediately frozen in liquid nitrogen, for storage at -80 °C until subsequent analyses (Fig. 5B and 5C). Taxonomic attribution of the trees used in this study was confirmed using diagnostic SNP markers described by [112].

A formal permission both from the National Park of the Pyrenees and to the different town halls was obtained in order to sample buds in the different populations used in this study.

### Quantification of hormones

For hormone quantification, we used three pools of six individuals each, corresponding to three biological replicates for each population and dormancy stage (i.e. 36 samples in total). Buds were independently ground to a fine powder in liquid nitrogen and freeze dried, and then mixed in equimolar ratios to constitute pools. Phytohormones were quantified as previously described [113].

### RNA extraction and sequencing

RNA was extracted independently for each individual and dormancy stage, according to the procedure described by [114]. Residual genomic DNA was removed before purification with DNase RQ1 (Promega, Madison, WI, USA), according to the manufacturer’s instructions. The quantity and quality of each extract were then determined with an Agilent 2100 Bioanalyzer (Agilent Technologies, Inc., Santa Clara, CA, USA). We generated 24 libraries, corresponding to 2 dormancy phases × 3 populations × 2 valleys × 2 biological replicates. For each biological replicate, we pooled equimolar amounts of RNA extracted from 10 individual trees. Libraries were generated with the Illumina protocol (TruSeq Stranded mRNA Sample Prep Kit, Illumina, San Diego, CA, USA). Briefly, we selected mRNA from a 2 µg sample of total RNA. mRNA was chemically fragmented and reversed transcribed with random hexamer primers. The second-strand cDNA was generated to create double-stranded cDNA. The cDNA was 3’-adenylated, and Illumina adapters were added. We amplified DNA fragments (with adapters) by PCR with Illumina adapter-specific primers. We quantified the libraries with a Qubit Fluorometer (Life Technologies, NY, USA). We estimated library size with Agilent 2100 Bioanalyzer technology (Agilent). We then sequenced each library by 101 base-length read chemistry, in a paired-end flow cell, on an Illumina HiSeq2000 sequencer (Illumina).

### Trimming, mapping and identification of differentially expressed genes (DEG)

Cleaning and mapping were performed as described by [115]. Briefly, we first removed low-quality reads and retained sequences with a mean quality value above 20. We mapped reads onto the 25,808 oak gene models published with the reference oak genome [116], using BWA (V.0.6.1) with the default parameters. We then identified differentially expressed genes (DEG) with the DESeq2 package, using a *P-*value < 0.01 after adjustment for multiple testing with a false discovery rate (FDR) of 5%. We also considered two-fold changes in expression ratio as a threshold for identifying the genes with the highest degree of differential expression. We investigated the effects of dormancy stage, elevation and their interaction in likelihood ratio tests. The dormancy and elevation effects were assessed by comparing a statistical model without interaction (M1) with two reduced models for the dormancy (M2) and elevation (M3) effects, respectively.

$$\mathrm{M}1:{\mathrm{Y}}_{\mathrm{ijk}}=\upmu +{\mathrm{D}}_{\mathrm{i}}+{\mathrm{E}}_{\mathrm{j}}+{\upvarepsilon }_{\mathrm{ijk}}$$

where D_i_ is the dormancy stage (i = ”Endodormancy” or “Ecodormancy”), and E_j_ the elevation effect (j = “low-100 m”, “medium-800 m” or “high-1,600 m” elevation).

$$\mathrm{M}2:{\mathrm{Y}}_{\mathrm{jk}}=\upmu +{\mathrm{E}}_{\mathrm{j}}+{\upvarepsilon }_{\mathrm{jk}}$$

$$\mathrm{M}3:{\mathrm{Y}}_{\mathrm{jk}}=\upmu +{\mathrm{D}}_{\mathrm{i}}+{\upvarepsilon }_{\mathrm{jk}}$$

For estimation of the interaction effect, we compared the following a complete model:

$${\mathrm{Y}}_{\mathrm{ijk}}=\upmu +{\mathrm{D}}_{\mathrm{i}}+{\mathrm{E}}_{\mathrm{j }}+ {\left(\mathrm{D}*\mathrm{E}\right)}_{\mathrm{ij }}+{\upvarepsilon }_{\mathrm{ijk}} \left(\mathrm{M}4\right)\; \mathrm{to}\;\mathrm{M1}$$

Three gene sets were thus generated: (i) DEGs (geneset#1) corresponding to dormancy regulation (regardless of elevation), (ii) DEGs (geneset#2) corresponding to differences in elevation (regardless of dormancy stage), and (ii) DEG (geneset#3) displaying a significant dormancy-by-elevation interaction. Annotations for each DEG were recovered from the published pedunculate oak genome sequence [116]. Below, we focus particularly on geneset#2 and #3, which should include the key molecular players involved in response to temperature (and potentially to local adaptation) to temperature for geneset#2, and should reveal differences in the strategies of oak stands analysed across bud phenological stages to cope with temperature variation for geneset#3. The elevation term used here does not allow to disentangle the effect of phenotypic plasticity from those of genetic differentiation. Indeed, [117] reported that the adaptive response of populations to divergent selection pressures along the elevation systematically results from a combination of non-optimal phenotypic plasticity and genetic differentiation. Thus, other experimental design such as reciprocal transplantations are needed to separate these two effects in the stands analyzed.

DEGs from geneset#2 and #3 were analyzed with EXPANDER software [118], which clusters genes according to their expression profile, using a Kmeans algorithm [118]. For both gene sets, we set the number of clusters to 5 (*k* = 5) to maximize the homogeneity of each cluster. The genes from each cluster were then used for independent gene set and subnetwork enrichment analysis (see below).

### Gene set and subnetwork enrichment analysis

GO term enrichment analyses were first performed with the TopGO R package [119, 120]. We considered corrected *P*-values below 0.05 (after adjustment for multiple testing with a false discovery rate (FDR of 5%)) to indicate significant enrichment in a particular GO term in our dataset. We then performed subnetwork enrichment analysis with Pathway Studio software (Pathway Studio®, Elsevier 2017), as described by [115].

### RT-qPCR validation

For the elevation and dormancy-by-elevation effects, we selected six genes each for RT-qPCR validation. All 12 genes analyzed are listed, together with their associated effect, in Additional File2 Table S3. RT-qPCR quantification was performed with 1 µg total RNA, as described by [121], on a Lightcycler® LC 384 (Roche Lifescience, Germany, EU) with standard parameters, as described by [115]. Fluorescence data were normalized against two control genes (Qrob_P0530610 and Qrob_P0426000) and analyzed by STATQPCR (http://satqpcr.sophia.inra.fr/cgi/home.cgi). All the primer pairs used here were designed with Primer3 software [122].

## Supplementary Information

**Additional file 1: Figure S1.** Evolution phytohormone content in each population according to the Dormancy stage. Panel A: Table for ANOVA results for phytohormonre analysis. *P* value is indicated in each cell. *P value<0.05, **P value<0.01 and ***P value<0.0001. NS stands for not significant. Panel B: Graphical representation of their accumulation over the Dormancy period. We used blue and orange color for EndoD (i.e. Endormancy) and EcoD (i.e. Ecodormancy) samples, respectivelly. Standard deviations were obtained from the 3 measurements performed in each population (Low=O-01+L-01, Mean=O-08+L-08 and High=O-18+L-18). Effects identified in the linear model where also indicated. Abbreviations correspond to: D: Dormancy effect, E: Elevation effect, D*E: interaction effect. * *P* value<0.05, **P value<0.001 and ***P value<0.0001). **Figure S2.** qPCR validation. Panel (**A**) genes displaying a significant elevation effect. Panel (**B**) genes displaying a significant Dormancy-byelevation effect. Abbreviations correspond to EndoD: Endodormant buds , EcoD: Ecodormant buds, Low: 100 mts (i.e. O-01+L-01), Mean: 800 mts (i.e. O-08+L-08) and High: 1,600 mts (i.e. O-16+L-16). Standard deviations were obtained from the four biological replicates.

**Additional file 2: Table S1. **Overview of the cDNA libraries generated in this study. **Table S2.** Gene expression level comparison between the two valleys for a specific dormancy stage. The comparison was performed for the population harvested at a same elevation in the two valleys considered. In each cell, we indicated the number of differentially expressed genes; the percentage is indicated in parenthesis. **Table S3.** List of the primer pairs used for qPCR analysis. Abbreviations: Tm: annealing temperature, E: Elevation effect, D*E: Dormancy-byelevation effect. For each effect, the cluster ID is indicated in parenthesis in the second column. Qrob_IDs were retrieved from the oak genome available in Plomion et al. (2018). **Table S4.** Overview of the sessile oak populations used in this study.

**Additional file 3: Supplementary Files 1, 2, 3. ** are available online at the INRAE dataverse portail: G. Le Provost, 2021^:“^Oak stands along an elevation gradient have different molecular strategies for regulating bud phenology” https://doi.org/10.15454/XMEKFX - Portail Data INRAE, V3.0. These files includes normalized values for RNAseq data, Fold change Ratio, Gene set enrichment analysis and subnetwork enrichment analysis for genes displaying a significant dormancy, elevation and dormancy-by-elevation interaction effect respectively.

## Data Availability

All the sequences generated for this publication have been deposited in the short-read archive of NCBI (https://www.ncbi.nlm.nih.gov/sra) under accession PRJEB17876. All data generated or analysed during this study are included in this published article and its supplementary online information files (Additional File 1 and 2 and Supplementary File 1,2 and 3).

## Abbreviations

EndoD: Endodormant buds
EcoD: Ecodormant buds
L: Luz valley
O: Ossau valley
O (L)-01: Ossau (or Luz) valley 100 m a.s.l
O (L)-08: Ossau (or Luz) valley 800 m a.s.l
O (L)-16: Ossau (or Luz) valley 1,600 m a.s.l
RT-qPCR: Quantitative Real Time Polymerase Chain Reaction
GO: Gene Ontology and
PCA: Principal Component Analysis
