# Supplementary material for: Oak stands along an elevation gradient have different molecular strategies for regulating bud phenology
Source: BMC Plant Biol. 2023 Feb 23;23:108. doi: 10.1186/s12870-023-04069-2 (PMC9948485; doi:10.1186/s12870-023-04069-2)
Supplement: Supplementary file 1 — Additional file 1: Figure S1. Evolution phytohormone content in each population according to the Dormancy stage. Panel A: Table for ANOVA results for phytohormonre analysis. P value is indicated in each cell. *P value<0.05, **P value<0.01 and ***P value<0.0001. NS stands for not significant. Panel B: Graphical representation of their accumulation over the Dormancy period. We used blue and orange color for EndoD (i.e. Endormancy) and EcoD (i.e. Ecodormancy) samples, respectivelly. Standard deviations were obtained from the 3 measurements performed in each population (Low=O-01+L-01, Mean=O-08+L-08 and High=O-18+L-18). Effects identified in the linear model where also indicated. Abbreviations correspond to: D: Dormancy effect, E: Elevation effect, D*E: interaction effect. * P value<0.05, **P value<0.001 and ***P value<0.0001). Figure S2. qPCR validation. Panel (A) genes displaying a significant elevation effect. Panel (B) genes displaying a significant Dormancy-byelevation effect. Abbreviations correspond to EndoD: Endodormant buds , EcoD: Ecodormant buds, Low: 100 mts (i.e. O-01+L-01), Mean: 800 mts (i.e. O-08+L-08) and High: 1,600 mts (i.e. O-16+L-16). Standard deviations were obtained from the four biological replicates. [file 12870_2023_4069_MOESM1_ESM.pdf]

Figure S1: Evolution phytohormone content in each population according to the Dormancy stage. Panel A: Table for ANOVA results for phytohormonre analysis. P value is indicated in each cell. *Pvalue<0.05, **Pvalue<0.01 and ***Pvalue<0.0001. NS stands for not significant. Panel B: Graphical representation of their accumulation over the Dormancy period. We used blue and orange color for EndoD (i.e. Endormancy) and EcoD (i.e. Ecodormancy) samples, respectivelly. Standard deviations were obtained from the 3 measurements performed in each population (Low=O-01+L-01, Mean=O-08+L-08 and High=O-18+L-18). Effects identified in the linear model where also indicated. Abbreviations correspond to: D: Dormancy effect, E: Elevation effect, D*E: interaction effect. * Pvalue<0.05, **Pvalue<0.001 and ***Pvalue<0.0001).

(A)

	IAA	ABA	Cytokinines
Dormancy	***<br>**$2.92\ 10^{-6}$**	***<br>**0.000015**	**<br>**0.00439**
Valley	NS<br>0.640	NS<br>0.653	NS<br>0.253
Elevation	NS<br>0.735	**<br>**0.005**	NS<br>0.174

(B)

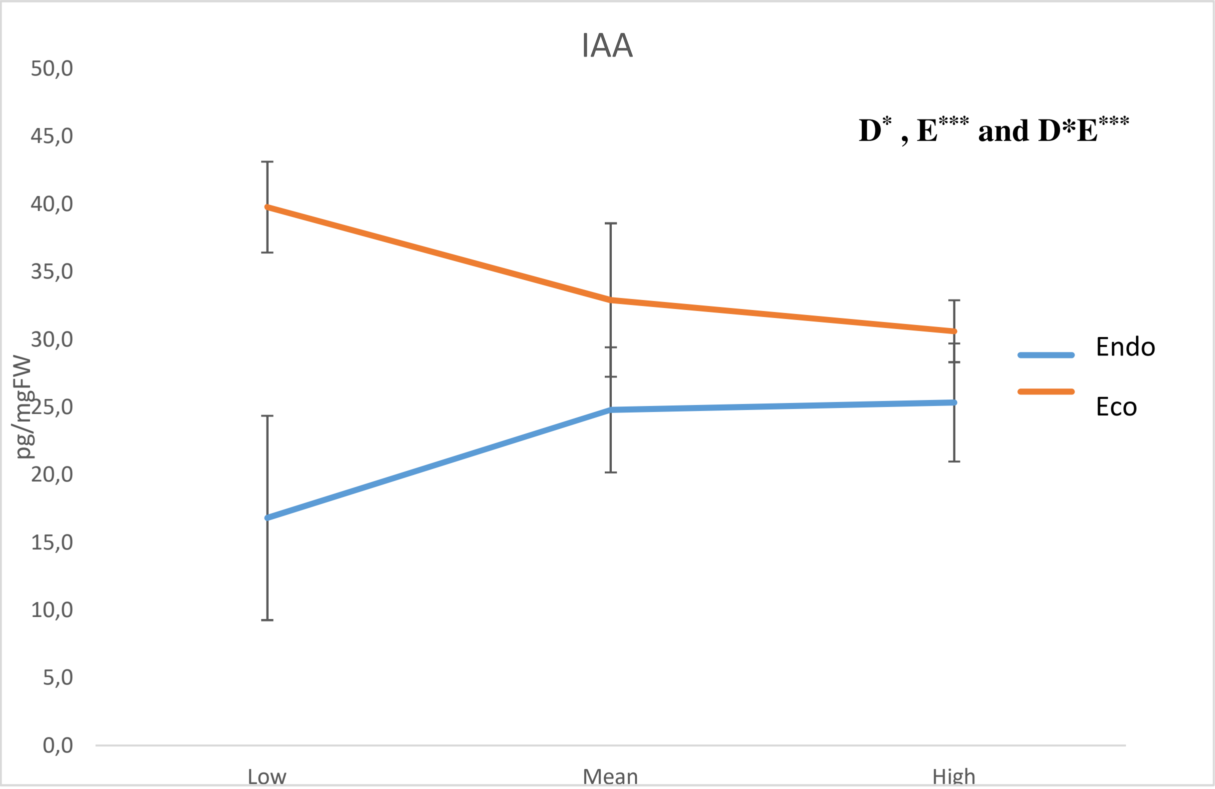

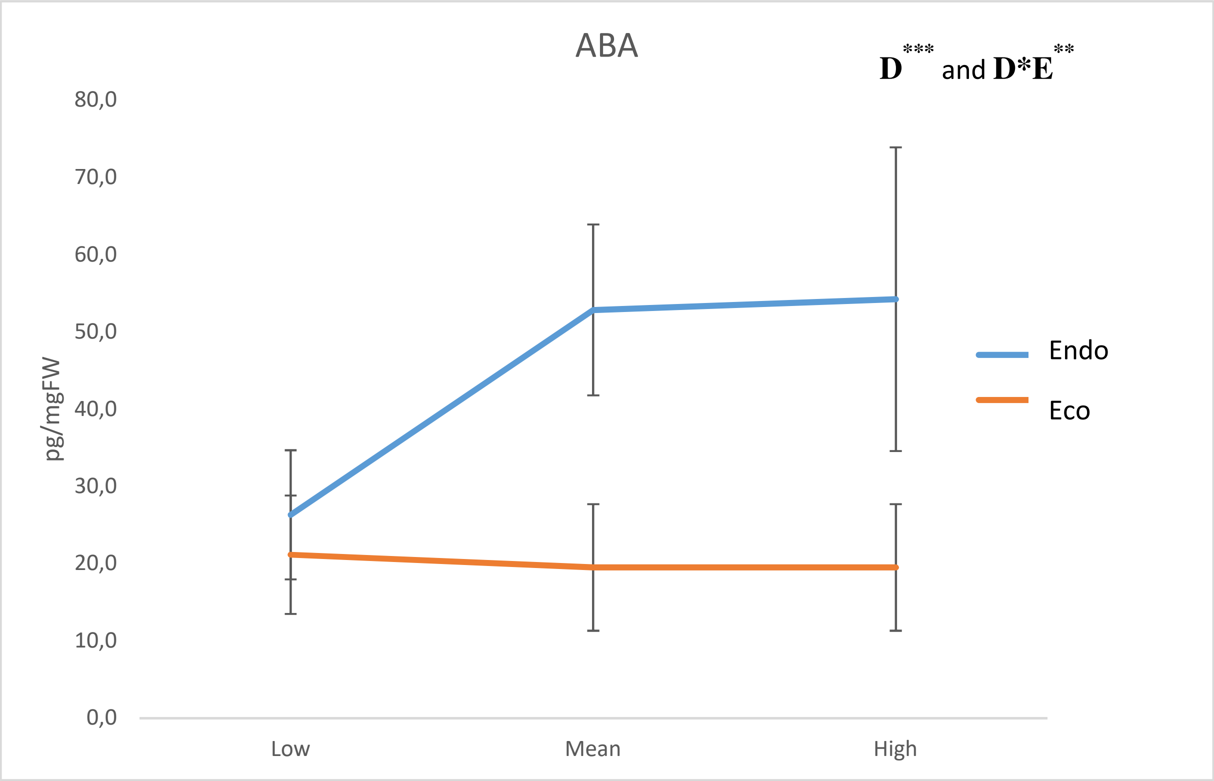
ABA
D*** and D*E**
80,0
70,0
60,0
50,0
40,0
30,0
20,0
10,0
0,0
pg/mgFW
Low
Mean
High
Endo
Eco

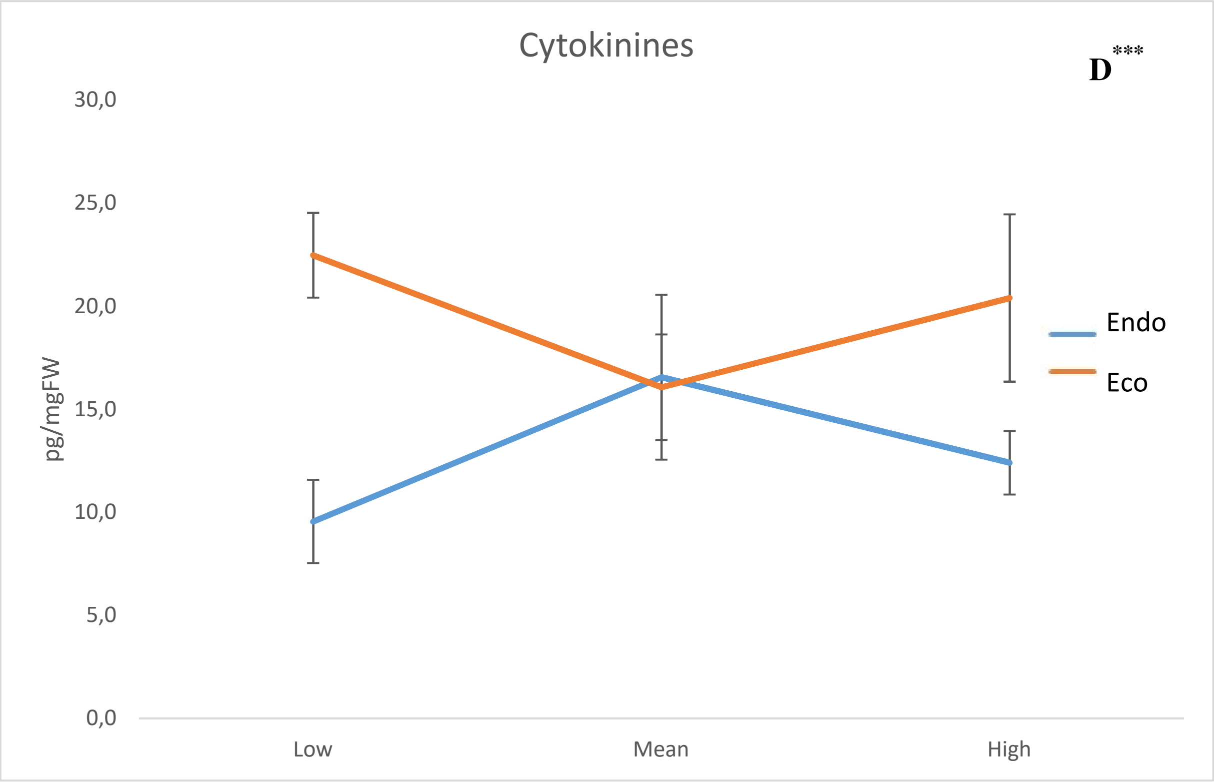
Cytokinines
D***
30,0
25,0
20,0
15,0
10,0
5,0
0,0
pg/mgFW
Low
Mean
High
Endo
Eco

Figure S2: qPCR validation. Panel (A) genes displaying a significant elevation effect. Panel (B) genes displaying a significant Dormancy-by-elevation effect. Abbreviations correspond to EndoD: Endodormant buds , EcoD: Ecodormant buds, Low: 100 mts (i.e. O-01+L-01), Mean: 800 mts (i.e. O-08+L-08) and High: 1,600 mts (i.e. O-16+L-16). Standard deviations were obtained from the four biological replicates

(A)

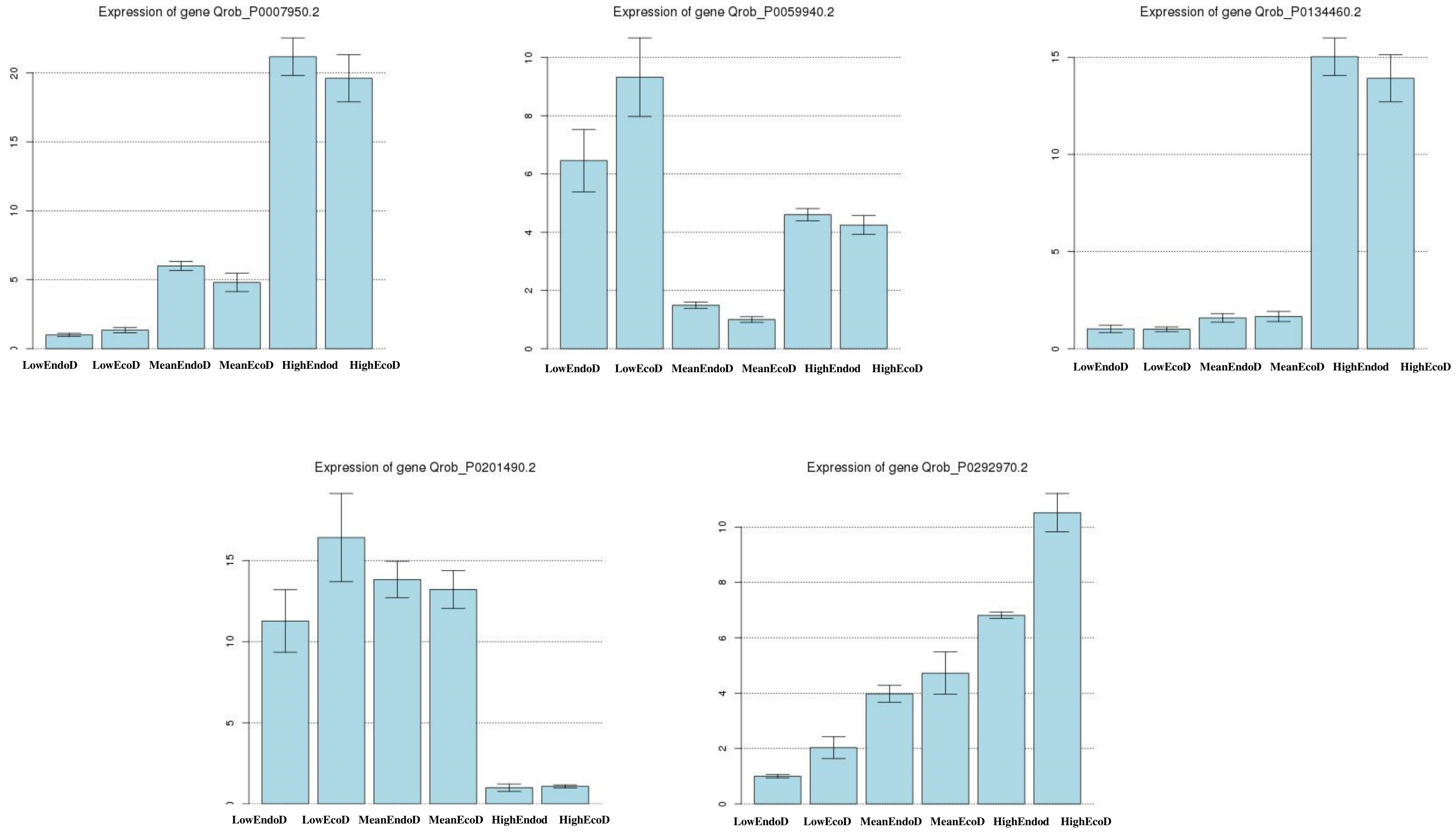

(B)

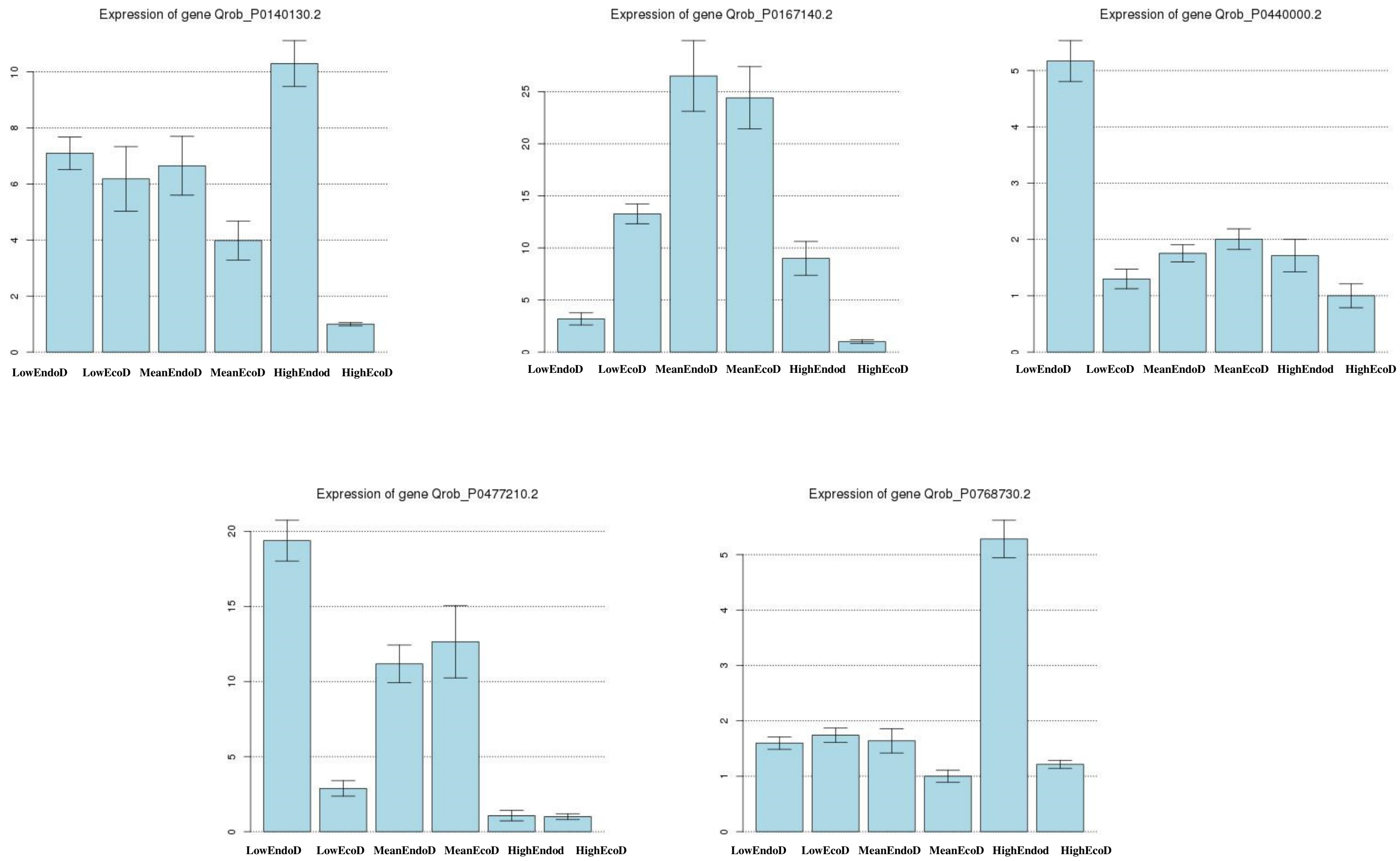

Expression of gene Qrob_P0140130.2
0
2
4
6
8
10
LowEndoD LowEcoD MeanEndoD MeanEcoD HighEndod HighEcoD
Expression of gene Qrob_P0167140.2
0
5
10
15
20
25
LowEndoD LowEcoD MeanEndoD MeanEcoD HighEndod HighEcoD
Expression of gene Qrob_P0440000.2
0
1
2
3
4
5
LowEndoD LowEcoD MeanEndoD MeanEcoD HighEndod HighEcoD
Expression of gene Qrob_P0477210.2
0
5
10
15
20
LowEndoD LowEcoD MeanEndoD MeanEcoD HighEndod HighEcoD
Expression of gene Qrob_P0768730.2
0
1
2
3
4
5
LowEndoD LowEcoD MeanEndoD MeanEcoD HighEndod HighEcoD
