# Supplementary material for: Oak stands along an elevation gradient have different molecular strategies for regulating bud phenology
Source: BMC Plant Biol. 2023 Feb 23;23:108. doi: 10.1186/s12870-023-04069-2 (PMC9948485; doi:10.1186/s12870-023-04069-2)
Supplement: Supplementary file 2 — Additional file 2: Table S1. Overview of the cDNA libraries generated in this study. Table S2. Gene expression level comparison between the two valleys for a specific dormancy stage. The comparison was performed for the population harvested at a same elevation in the two valleys considered. In each cell, we indicated the number of differentially expressed genes; the percentage is indicated in parenthesis. Table S3. List of the primer pairs used for qPCR analysis. Abbreviations: Tm: annealing temperature, E: Elevation effect, D*E: Dormancy-byelevation effect. For each effect, the cluster ID is indicated in parenthesis in the second column. Qrob_IDs were retrieved from the oak genome available in Plomion et al. (2018). Table S4. Overview of the sessile oak populations used in this study. [file 12870_2023_4069_MOESM2_ESM.pdf]

Table S1: Overview of the cDNA libraries generated in this study.

Library	Dormancy stage	Valley	Elevation in meters	Biological replicate	Number of read	Number of read mapped	% of mapped read	ENA study Accesion
Josbaig 1 EndoD (L-01)	EndoD	Luz	100	Bio. Rep. 1	33,041,456	26,108,308	78%	PRJEB17876
Josbaig 2 EndoD (L-01)	EndoD	Luz	100	Bio. Rep. 2	39,872,577	30,912,308	76%	
Josbaig 1 EcoD (L-01)	EcoD	Luz	100	Bio. Rep. 1	50,981,819	41,289,518	81%	
Josbaig 2 EcoD (L-01)	EcoD	Luz	100	Bio. Rep. 2	23,561,827	19,087,016	82%	
Le Hourque 1 EndoD (L-08)	EndoD	Luz	800	Bio. Rep. 1	30,616,326	23,634,328	76%	
Le Hourque 2 EndoD (L-08)	EndoD	Luz	800	Bio. Rep. 2	29,858,750	23,458,930	79%	
Le Hourque 1 EcoD (L-08)	EcoD	Luz	800	Bio. Rep. 1	27,938,013	21,945,696	77%	
Le Hourque 2 EcoD (L-08)	EcoD	Luz	800	Bio. Rep. 2	26,431,779	21,060,600	80%	
Papillon 1 EndoD (O-08)	EndoD	Ossau	800	Bio. Rep. 1	28,255,115	22,242,856	78%	
Papillon 2 EndoD (O-08)	EndoD	Ossau	800	Bio. Rep. 2	28,507 89	22,407,716	78%	
Papillon 1 EcoD (O-08)	EcoD	Ossau	800	Bio. Rep. 1	24,630,650	16,888,852	66%	
Papillon 2 EcoD (O-08)	EcoD	Ossau	800	Bio. Rep. 2	30,949,276	22,751,666	73%	
Peguere 1 EndoD (O-16)	EndoD	Ossau	1,600	Bio. Rep. 1	38,381,974	28,196,854	73%	
Peguere 2 EndoD (O-16)	EndoD	Ossau	1,600	Bio. Rep. 2	33,474,713	25,096,294	75%	
Peguere 1 EcoD (O-16)	EcoD	Ossau	1,600	Bio. Rep. 1	35,260,832	25,899,890	71%	
Peguere 2 EcoD (O-16)	EcoD	Ossau	1,600	Bio. Rep. 2	37,076,987	27,232,020	72%	
Artouste 1 EndoD (L16)	EndoD	Luz	1,600	Bio. Rep. 1	33,963,824	26,114,606	78%	
Artouste 2 EndoD (L16)	EndoD	Luz	1,600	Bio. Rep. 2	32,241,220	25,563,258	78%	
Artouste 1 EcoD (L16)	EcoD	Luz	1,600	Bio. Rep. 1	28,924,891	22,514,446	78%	
Artouste 2 EcoD (L16)	EcoD	Luz	1,600	Bio. Rep. 2	28,146,228	20,581,192	71%	

**Laveyron 1 EndoD (O-01)**	**EndoD**	**Ossau**	**100**	**Bio. Rep. 1**	32,668,746	25,573,700	78%	
**Laveyron 2 EndoD (O-01)**	**EndoD**	**Ossau**	**100**	**Bio. Rep. 2**	29,920,489	23,661,520	79%	
**Laveyron 1 EcoD (O-01)**	**EcoD**	**Ossau**	**100**	**Bio. Rep. 1**	33,368,451	27,291,694	81%	
**Laveyron 2 EcoD (O-01)**	**EcoD**	**Ossau**	**100**	**Bio. Rep. 2**	33,982,054	27,638,362	81%	

Table S2: Gene expression level comparison between the two valleys for a specific dormancy stage. The comparison was performed for the population harvested at a same elevation in the two valleys considered. In each cell, we indicated the number of differentially expressed genes; the percentage is indicated in parenthesis.

	EndoD	EcoD
O-01 vs. L-01	76<br>(0.6%)	81<br>(0.6%)
O-08 vs. L-08	149<br>(1.2%)	78<br>(0.6%)
O-16 vs. L-16	55<br>(0.4%)	93<br>(0.7%)

Table S3: List of the primer pairs used for qPCR analysis. Abbreviations: Tm: annealing temperature, E: Elevation effect, D*E: Dormancy-by-elevation effect. For each effect, the cluster ID is indicated in parenthesis in the second column. Qrob_IDs were retrieved from the oak genome available in Plomion et al. (2018).

.

Gene ID	*Significant effect in RNAseq*	*Function*	*Forward primer(5'3')*<br>*Reverse primer (3'-5')*	*Amplicon size (bp)*	*Multiband in agarose gel*	*Used in qPCR*	*PCR Efficiency*	*Highest expression in qPCR*
Qrob_P0007950.2	E (CL1)	**Disease resistance-responsive protein**	GCAATCCCTCTTCAGTCCCA<br>CACACTGAGTTCCTGCCTAG	276	No	Yes	103	**High elevation**
Qrob_P0292970.2	E (CL1)	**Unknown protein**	TGTTGTGATGAAGGCAGACG<br>ACAGCAACTCCCTCATCCAA	192	No	Yes	108	**High elevation**
Qrob_P0134460.2	E (CL4)	**Metal transport/detoxification superfamily protein**	GTCCAAGGCCATGCAGATTG<br>GTACTGACTGGCGAGACACT	177	No	Yes	95	**High elevation**
Qrob_P0247760.2	E(CL4)	**TIR-NBS-LRR**	GACGGCTTTCATGACCAACA<br>AATTTCTTCTCCCCTCGGCA	181	Yes	No	NA	**NA**
Qrob_P0059940.2	E(CL2)	**G-type lectin S-receptor-like serine/threonine-protein kinase**	GACACCATCTCTGCACACCA<br>GTCTCTCTGTTTGCCACCCA	179	No	yes	97	**Low elevation**
Qrob_P0201490.2	E(CL5)	**Urine biosynthesis 4 protein**	TTTTCCTCGCCCCAATAGGT<br>ATAGGGTGCGTCAGTTGGAA	244	No	yes	107	**Low/Mean elevation**
Qrob_P0440000.2	D*E (CL2)	**Sucrose synthase 3**	TTTTCCTCGCCCCAATAGGT<br>ATAGGGTGCGTCAGTTGGAA	214	No	yes	103	**D*E**
Qrob_P0477210.2	D*E (CL2)	**Pyrophosphorylase 2**	TGATCCTGAGTTCCGCCATT<br>GGCTTCAATGGCAGACTCAG	151	No	Yes	98	**D*E**
Qrob_P0252420.2	D*E (CL3)	**Amino Acid permease 7**	ACCTGATGAACCTGAGGAGC<br>TTCTAGCAGGGCCACATTCA	236	Yes	No	NA	**NA**
Qrob_P0140130.2	D*E (CL4)	**TCP domain protein 9**	GATGCAAGTGACGCCAGTAG<br>AGTTCTCGGGTCAGCTGAAA	171	No	Yes	98	**D*E**
Qrob_P0768730.2	D*E (CL4)	**GDP-D-mannose 4,6-**	AATTCAAGTGCTCCTCCGGT<br>GCTCTGCTTGTATCTCCCTT					**D*E**

		**dehydratase 1**		193	No	Yes	98	
Qrob_P0167140.2	D*E (CL5)	**Senescence Associated Gene 101 (SAG101)**	GGCAAATTTCGTGGTGACCC<br>TTGCTACTAGAGGGCCAGGT	171	No	Yes	99	**D*E**
			**Control Genes**					
Qrob_P0530610	NS	**Unknown protein**	GAAGCACCACCCTCACAAGT<br>GTCTCCTCACAACTCACCGG	197	No	Yes	110	**NA**
Qrob_P0426000	NS	**Unknown protein**	GCAGAGCTCCAGGACATGATA<br>CAGCAGCAGAGATGAACCCA	175	No	Yes	106	**NA**

Table S4: Overview of the sessile oak populations used in this study.

Populations	Valleys	Elevations (meters)	Latitude Longitude	Endodormancy sampling dates	Ecodormancy sampling dates
Laveyron (O-01)	Ossau	100	43°45’49” N 0° 13’11” W	6 October 2013	12 March 2014
Papillon (O-08)	Ossau	800	43° 25’ 23” N 0° 1’59” W	7 October 2013	17 March 2014
Péguere (O16)	Ossau	1,600	43° 52’ 00” N 0° 07’09” W	8 October 2013	7 April 2014
Josbaig (L-01)	Luz	100	42°13’35” N 0° 44’28” W	10 October 2013	10 March 2014
LeHourcq (L-08)	Luz	800	42°54’46” N 0° 26’04” W	10 October 2013	16 March 2014
Artouste (L16)	Luz	1,600	42°53’00” N 0° 24’08” W	9 October 2013	7 April 2014
