# Supplementary material for: Oak stands along an elevation gradient have different molecular strategies for regulating bud phenology
Source: BMC Plant Biol. 2023 Feb 23;23:108. doi: 10.1186/s12870-023-04069-2 (PMC9948485; doi:10.1186/s12870-023-04069-2)
Supplement: Supplementary file 3 — Additional file 3: Supplementary Files 1, 2, 3. are available online at the INRAE dataverse portail: G. Le Provost, 2021:“Oak stands along an elevation gradient have different molecular strategies for regulating bud phenology” https://doi.org/10.15454/XMEKFX - Portail Data INRAE, V3.0. These files includes normalized values for RNAseq data, Fold change Ratio, Gene set enrichment analysis and subnetwork enrichment analysis for genes displaying a significant dormancy, elevation and dormancy-by-elevation interaction effect respectively. [file 12870_2023_4069_MOESM3_ESM.docx]

**Supplementary Files 1, 2, 3** are available online at the INRAE dataverse portail: G. Le Provost, 2021^:“^Oak stands along an elevation gradient have different molecular strategies for regulating bud phenology” <https://doi.org/10.15454/XMEKFX> - Portail Data INRAE, V3.0. These files includes normalized values for RNAseq data, Fold change Ratio, Gene set enrichment analysis and subnetwork enrichment analysis for genes displaying a significant dormancy, elevation and dormancy-by-elevation interaction effect respectively.
